# The complete mitochondrial genome of *Sesarmops sinensis* reveals gene rearrangements and phylogenetic relationships in Brachyura

**DOI:** 10.1371/journal.pone.0179800

**Published:** 2017-06-16

**Authors:** Bo-Ping Tang, Zhao-Zhe Xin, Yu Liu, Dai-Zhen Zhang, Zheng-Fei Wang, Hua-Bin Zhang, Xin-Yue Chai, Chun-Lin Zhou, Qiu-Ning Liu

**Affiliations:** Jiangsu Key Laboratory for Bioresources of Saline Soils, Jiangsu Synthetic Innovation Center for Coastal Bio-agriculture, Jiangsu Provincial Key Laboratory of Coastal Wetland Bioresources and Environmental Protection, School of Ocean and Biological Engineering, Yancheng Teachers University, Yancheng, PR China; Zhejiang University College of Life Sciences, CHINA

## Abstract

Mitochondrial genome (mitogenome) is very important to understand molecular evolution and phylogenetics. Herein, in this study, the complete mitogenome of *Sesarmops sinensis* was reported. The mitogenome was 15,905 bp in size, and contained 13 protein-coding genes (PCGs), two ribosomal RNA (rRNA) genes, 22 transfer RNA (tRNA) genes, and a control region (CR). The AT skew and the GC skew are both negative in the mitogenomes of *S*. *sinensis*. The nucleotide composition of the *S*. *sinensis* mitogenome was also biased toward A + T nucleotides (75.7%). All tRNA genes displayed a typical mitochondrial tRNA cloverleaf structure, except for the *trnS1* gene, which lacked a dihydroxyuridine arm. *S*. *sinensis* exhibits a novel rearrangement compared with the Pancrustacean ground pattern and other Brachyura species. Based on the 13 PCGs, the phylogenetic analysis showed that *S*. *sinensis* and *Sesarma neglectum* were clustered on one branch with high nodal support values, indicating that *S*. *sinensis* and *S*. *neglectum* have a sister group relationship. The group (*S*. *sinensis + S*. *neglectum*) was sister to (*Parasesarmops tripectinis* + *Metopaulias depressus*), suggesting that *S*. *sinensis* belongs to Grapsoidea, Sesarmidae. Phylogenetic trees based on amino acid sequences and nucleotide sequences of mitochondrial 13 PCGs using BI and ML respectively indicate that section Eubrachyura consists of four groups clearly. The resulting phylogeny supports the establishment of a separate subsection Potamoida. These four groups correspond to four subsections of Raninoida, Heterotremata, Potamoida, and Thoracotremata.

## Introduction

The Brachyura mostly live in littoral regions of tropical shallow seas and about 7000 species have been described and is the most species rich infraorder within Decapoda [1]. Phylogenetic relationships within the Brachyura are complicated because of the extreme morphological and ecological diversity within the group [2]. Four groups of Brachyura (Dromiacea, Raninoida, Heterotremata and Thoracotremata) were recognized [3–5]. Raninoida, Heterotremata and Thoracotremata should be attributed to Eubrachyura, which is a sisiter group to Dromiacea [6,7].

Animal mitochondrial DNA (mtDNA), a double-stranded circular molecule, ranging from 14 to 19 kilobases (kb) in size, containing 37 genes, including 13 PCGs, ATPase subunits 6 and 8 of the ATPase (*atp6* and *atp8*), cytochrome c oxidase subunits 1–3 (*cox1*–*cox3*), cytochrome B (*cob*), NADH dehydrogenase subunits 1–6 and 4 L (*nad1*–*6* and *nad4L*), 22 tRNA genes, two rRNA genes and CR [8]. The mtDNA can provide important information on rearrangement trends and phylogeny because of its rapid evolutionary rate and lack of genetic recombination [8]. Using complete mitogenomes is becoming increasingly common for phylogenetic reconstruction [9–11]. The AT-content, secondary structures of tRNAs, and gene rearrangements can be inferred from animal mitogenomes at the genome level [12]. Partial DNA sequences are often too short to contain sufficient phylogenetic information [13]. Further, the addition of rRNA makes alignment ambiguous [2]. It is becoming increasingly common to use complete animal mitogenomes for phylogenetic reconstruction [9–11].

To date, there has been no reports of the complete mitogenome of *S*. *sinensis*. Thus, in this paper, the complete mitogenome of *S*. *sinensis* was sequenced and compared with other Brachyura mitogenomes. The available complete mitogenomes were used to provide insight into the phylogenetic relationship of *S*. *sinensis* and related species. These results will help us to understand features of *S*. *sinensis* mitogenome and the evolutionary relationships within Brachyura.

## Materials and methods

### Ethics statement

There are no specific permits for crabs collection in the selected locations. The sampling locations are not privately-owned or natural protected areas. Crabs used for the experiments are not considered endangered or protected species, and its collection is legal in China.

### Sampling and DNA extraction

Adult specimens of *S*. *sinensis* were collected from Fujian province in China in June 2014. The total genomic DNA of *S*. *sinensis* was isolated from single specimens using the Aidlab Genomic DNA Extraction Kit (Aidlab, China) according to the manufacturer’s instructions. DNA from an individual *S*. *sinensis* crab was used to amplify the complete mitogenome.

### PCR amplification and sequencing

To amplify the entire mitogenome of *S*. *sinensis*, specific primers were designed based on the conserved nucleotide sequences of known mitochondrial sequences in the Brachyura [14–18]. The complete mitogenome of *S*. *sinensis* was obtained using a combination of conventional PCR and long PCR to amplify overlapping fragments spanning the whole mitogenome. First, six conserved genes (*cox1*, *cox3*, *12S*, *16S*, *nad4*, *cob*) were sequencd using the universal primer of crabs. Then the specific primers were designed by conservative sequences. Finally the complete mitogenome was generated by overlapping PCR with specific primers. The fragments were amplified using Aidlab Red Taq (Aidlab) according to the manufacturer’s instructions. PCR reactions for the fragments were performed in a 50μL volume with 5μL of 10×Taq plus Buffer (Mg^2+^), 4μL of dNTPs, 2μL each of the primers, 2μL of DNA, 34.5μL of ddH_2_O, and 0.5μL Red Taq DNA polymerase. The PCR reactions were performed using the following procedures: 94°C for 3 min; followed by 40 cycles of 30s at 94°C, annealing for 35s at 48–56°C (depending on primer combination), elongation for 1–3min (depending on length of the fragments) at 72°C; and a final extension step of 72°C for 10 min. The PCR products were separated by agarose gel electrophoresis (1% w/v) and purified using the DNA gel extraction kit (Aidlab, China). The purified PCR products were ligated into the T-vector (SangonBiotech, China) and sequenced at least three times.

### Sequence alignment and gene annotation

Thirty-nine complete Brachyura mitogenomes were downloaded from GenBank. In addition, the mitogenomes of *Cherax destructor* and *Cambaroides similis* were downloaded from GenBank and used as outgroup taxons. Detailed information is shown in Table 1.

**Table 1:** List of 40 Brachyura species analysed in this paper with their GenBank accession numbers.

Species	Family	Size (bp)	Accession No.
*Sesarmops sinensis*	Sesarmidae	15,905	KR336554
*Sesarma neglectum*	Sesarmidae	15,920	KX156954
*Metopaulias depressus*	Sesarmidae	15,765	KX118277
*Parasesarmops tripectinis*	Sesarmidae	15,612	KU343209
*Helice latimera*	Varunidae	16,246	KU589291
*Eriocheir japonica sinensis*	Varunidae	16,378	KM516908
*Eriocheir japonica hepuensis*	Varunidae	16,335	FJ455506
*Eriocheir japonica japonica*	Varunidae	16,352	FJ455505
*Cyclograpsus granulosus*	Varunidae	16,300	LN624373
*Gandalfus yunohana*	Bythograeidae	15,567	EU647222
*Gandalfus puia*	Bythograeidae	15,548	KR002727
*Austinograea alayseae*	Bythograeidae	15,620	JQ035660
*Austinograea rodriguezensis*	Bythograeidae	15,611	JQ035658
*Ocypode cordimanus*	Ocypodidae	15,604	KT896743
*Ocypode ceratophthalmus*	Ocypodidae	15,564	LN611669
*Umalia orientalis*	Raninidae	15,466	KM365084
*Lyreidus brevifrons*	Raninidae	16,112	KM983394
*Homologenus malayensis*	Homolidae	15,793	KJ612407
*Moloha majora*	Homolidae	15,903	KT182069
*Pseudocarcinus gigas*	Menippidae	15,515	AY562127
*Myomenippe fornasinii*	Menippidae	15,658	LK391943
*Geothelphusa dehaani*	Potamidae	18,197	AB187570
*Huananpotamon lichuanense*	Potamidae	15,380	KX639824
*Portunus pelagicus*	Portunidae	16,157	KM977882
*Callinectes sapidus*	Portunidae	16,263	AY363392
*Portunus trituberculatus*	Portunidae	16,026	AB093006
*Portunus sanguinolentus*	Portunidae	16,024	KT438509
*Charybdis japonica*	Portunidae	15,738	FJ460517
*Scylla paramamosain*	Portunidae	15,824	JX457150
*Scylla olivacea*	Portunidae	15,723	FJ827760
*Scylla tranquebarica*	Portunidae	15,833	FJ827759
*Scylla serrata*	Portunidae	15,775	FJ827758
*Charybdis feriata*	Portunidae	15,660	KF386147
*Pachygrapsus crassipes*	Grapsidae	15,652	KC878511
*Ilyoplax deschampsi*	Dotillidae	15,460	JF909979
*Damithrax spinosissimus*	Mithracidae	15,817	KM405516
*Macrophthalmus japonicus*	Macrophthalmidae	16,170	KU343211
*Dynomene pilumnoides*	Dynomenidae	16,475	KT182070
*Xenograpsus testudinatus*	Xenograpsidae	15,798	EU727203
*Mictyris longicarpus*	Mictyridae	15,548	LN611670

Sequence annotation was performed using NCBI BLAST and the DNAStar package (DNAStar Inc. Madison, WI, USA). Alignments of sequences for each of the available Brachyura mitogenomes were performed using default settings in MAFFT, and then concatenated [19]. The sequences were aligned with those of closely related species. To remove the gaps in sequences and align each gene, poorly aligned positions and divergent regions were removed using Gblocks in our study [20]. The fasta sequences were converted to the nex format and phylip format for BI and ML, respectively. We then used DAMBE to detect the saturated conditions of the sequences [21]; the result of the DAMBE analysis was that ISS was less than ISS.c and p value was extremely significant (0.0000), suggesting that sequences was unsaturated and suit to construct phylogenetic tree. Cloverleaf secondary structure and anticodons of transfer RNAs were identified using the web-server of tRNA-scan SE [22].

### Phylogenetic analysis

We estimated the taxonomic status of *S*. *sinensis* within the Decapoda by constructing the phylogenetic tree. Two concatenated datasets: amino acid alignments (AA dataset) and nucleotide alignments (NT dataset) from 42 mitogenomes PCGs were combined. Each dataset was processed using two inference methods: Bayesian inference (BI) and Maximum likelihood (ML). BI was performed using MrBayes v 3.2.1 [23]. ML was performed using raxmlGUI [24]. Nucleotide substitution models were selected using Akaike information criterion implemented in Mrmodeltest v 2.3 [25]. The GTR+I+G model was chosen as the best model of nucleotide phylogenetic analysis and molecular evolution. MtArt + I+ G +F was chosen as the best model for the AA dataset, according to the results of Prottest version 1.4 [26]. There was no MtArt + I+ G +F model in MrBayes; therefore, MtREV + I+ G +F was selected as the second best model. ML analyses were performed on 1000 bootstrapped replicates [27]. The Bayesian analysis ran as 4 simultaneous MCMC chains for 10,000,000 generations, sampled every 100 generations, and a burn-in of 2,500,000 generations was used. Convergence was deduced for the Bayesian analysis on the following basis: the average standard deviation of split frequencies was less than 0.01. Additionally, we observe sufficient parameter sampling by using software Tracer v1.6. The value of ESS is more than 200. The above two points show that our data is convergent [28]. BI was performed under the GTRCAT model with the NT dataset. ML was performed with ML+rapid bootstrap under the GTRCAT model with the NT dataset. BI and ML were performed under the MtREV + I+ G +F model with the AA dataset.

## Results and discussion

### Genome structure and organization

**Fig 1:**
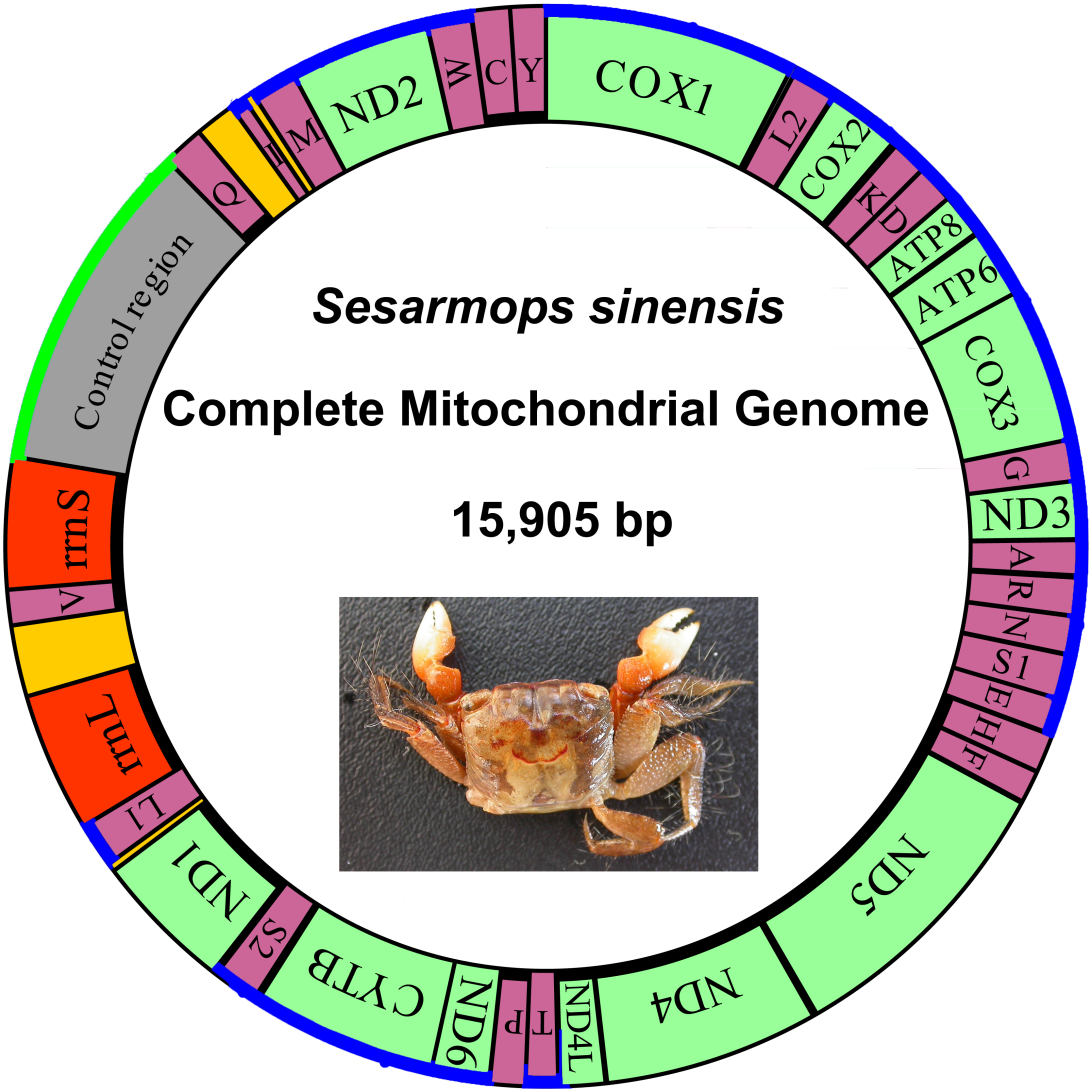
Circular map of the mitogenome of *S*. *sinensis*. Protein coding and ribosomal genes are shown with standard abbreviations. Genes for tRNAs are abbreviated by a single letter, with S1 = AGN, S2 = UCN, L1 = CUN, and L2 = UUR. 13 Protein coding genes are yellow colored. tRNAs are purple colored. rRNAs are red colored.

The *S*. *sinensis* mitogenome is a closed circular molecule of 15,905 bp in length. It has been deposited in GenBank under the accession number KR336554 and contains typical animal mitochondrial genes, including 13 PCGs, 22 tRNA genes, a large ribosomal RNA (lrRNA) gene and a small ribosomal RNA (srRNA) genes, and CR (Table 2 and Fig 1). Twenty-three genes are coded on the majority strand and the remaining fourteen genes are transcribed on the minority strand. The *S*. *sinensis* nucleotide composition is (A) 37.4%, (T) 38.3%, (G) 9.4%, and (C) 14.9%. It shows a high A+T bias: the A+T nucleotide content is 75.7%. In addition, the A+T skew value ([A–T]/[A+T]) is –0.012, and the G+C skew value ([G–C]/[G+C]) is –0.228 [29]. The AT skew and GC skew were calculated for the selected complete mitogenomes (Table 3). The A+T skew value is in the range from –0.080 (*Pachygrapsus crassipes*) to 0.040 (*Homologenus malayensis*). The GC skew values were negative in all sequenced Brachyura mitogenomes, ranging from –0.349 (*Macrophthalmus japonicus*) to –0.215 (*P*. *tripectinis*). Although the AT skew and GC skew of *S*. *sinensis* are all negative, GC skew is far lower than that of AT indicates an obvious bias toward the use of As and Cs.

**Table 2:** Summary of mitogenome of *S*. *sinensis*.

Gene	Direction	Location	Size	Anticodon	Start codon	Stop codon	Intergenic nucleotides
*cox1*	F	1–1559	1559	—	ATG	TAA	-24
*trnL2*	F	1536–1601	66	TAA	—	—	6
*cox2*	F	1608–2315	708	—	ATG	TAA	-20
*trnK*	F	2296–2365	70	TTT	—	—	-1
*trnD*	F	2365–2428	64	GTC	—	—	0
*atp8*	F	2429–2587	159	—	ATG	TAA	-7
*atp6*	F	2581–3254	674	—	ATG	TA	0
*cox3*	F	3255–4045	791	—	ATG	TA	0
*trnG*	F	4046–4110	65	TCC	—	—	0
*nad3*	F	4111–4461	351	—	ATT	TAA	4
*trnA*	F	4466–4529	64	TGC	—	—	10
*trnR*	F	4540–4605	66	TCG	—	—	2
*trnN*	F	4608–4674	67	GTT	—	—	3
*trnS1*	F	4678–4744	67	TCT	—	—	0
*trnE*	F	4745–4810	66	TTC	—	—	4
*trnH*	R	4815–4878	64	TAC	—	—	2
*trnF*	R	4881–4945	65	GAA	—	—	7
*nad5*	R	4953–6680	1728	—	ATG	TAA	19
*nad4*	R	6700–8061	1392	—	ATG	TAA	-7
*nad4L*	R	8055–8357	303	—	ATG	TAA	9
*trnT*	F	8367–8432	66	TGT	—	—	0
*trnP*	R	8433–8499	67	TGG	—	—	2
*nad6*	F	8502–9004	503	—	ATT	TA	0
*cob*	F	9005–10,159	1155	—	ATG	TAA	-20
*trnS2*	F	10,140–10206	67	TGA	—	—	19
*nad1*	R	10,226–11164	939	—	ATA	TAA	40
*trnL1*	R	11,205–11,268	64	TAG	—	—	0
*rrnL*	R	11,269–12,267	999	—	—	—	340
*trnV*	R	12,608–12680	73	TAC	—	—	0
*rrnS*	R	12,681–13,502	822	—	—	—	0
CR	—	13,503–14,253	751	—	—	—	0
*trnQ*	R	14,254–14,322	69	TTG	—	—	192
*trnI*	F	14,515–14,581	67	GAT	—	—	47
*trnM*	F	14,629–14,698	70	CAT	—	—	0
*nad2*	F	14,699–15,706	1008	—	ATG	TAG	2
*trnW*	F	15,709–15,778	70	TCA	—	—	-3
*trnC*	R	15,776–15,839	64	GCA	—	—	0
*trnY*	R	15,840–15,905	66	GTA	—	—	—

**Table 3:** The A+T skew value and the G+C skew value of 40 Brachyura species.

Species	Size (bp)	A %	G %	T %	C %	A+T %	A+T skew	G+C skew
*S*. *sinensis*	15,905	37.4	9.4	38.3	14.9	75.7	-0.012	-0.228
*H*. *latimera*	16,246	34.0	11.0	35.1	19.9	69.1	-0.017	-0.290
*G*. *puia*	15,548	35.1	10.3	34.8	19.8	69.9	0.006	-0.313
*P*. *sanguinolentus*	16,024	31.6	12.9	34.0	21.5	65.6	-0.037	-0.243
*E*. *j*. *sinensis*	16,378	35.2	10.8	36.4	17.6	71.6	-0.016	-0.243
*E*. *j*. *hepuensis*	16,335	35.1	10.8	36.4	17.7	71.5	-0.018	-0.245
*E*. *j*. *japonica*	16,352	35.2	10.7	36.5	17.7	71.7	-0.018	-0.245
*X*. *testudinatus*	15,798	36.7	9.3	37.2	16.8	73.9	-0.007	-0.297
*P*. *gigas*	15,515	35.0	10.8	35.5	18.7	70.5	-0.006	-0.268
*G*. *dehaani*	18,197	36.9	8.3	38.0	16.8	74.9	-0.014	-0.341
*L*. *brevifrons*	16,112	34.2	11.3	36.4	18.1	70.6	-0.031	-0.231
*C*. *sapidus*	16,263	34.2	11.1	34.9	19.8	69.1	-0.011	-0.279
*P*. *trituberculatus*	16,026	33.3	11.3	36.9	18.5	70.2	-0.051	-0.241
*H*. *malayensis*	15,793	37.3	10.0	34.4	18.3	71.7	0.040	-0.292
*C*. *japonica*	15,738	33.8	11.9	35.4	18.9	69.2	-0.024	-0.228
*S*. *paramamosain*	15,824	34.9	10.1	38.2	16.8	73.1	-0.045	-0.247
*U*. *orientalis*	15,466	33.1	11.8	34.9	20.2	68.0	-0.027	-0.262
*S*. *olivacea*	15,723	33.5	11.2	35.9	19.4	69.4	-0.035	-0.267
*S*. *tranquebarica*	15,833	35.0	9.8	38.7	16.5	73.7	-0.050	-0.258
*S*. *serrata*	15,775	34.5	10.4	38.0	17.1	72.5	-0.047	-0.242
*D*. *spinosissimus*	15,817	33.3	10.5	36.8	19.4	70.1	-0.050	-0.294
*C*. *feriata*	15,660	34.1	11.2	36.1	18.6	70.2	-0.028	-0.246
*G*. *yunohana*	15,567	34.3	10.8	35.6	19.3	69.9	-0.019	-0.281
*P*. *pelagicus*	16,157	33.7	12.2	35.0	19.1	68.8	-0.019	-0.219
*A*. *alayseae*	15,620	34.4	11.4	32.4	21.8	66.8	0.029	-0.316
*A*. *rodriguezensis*	15,611	35.3	10.3	33.5	20.9	68.8	0.025	-0.341
*P*. *crassipes*	15,652	30.5	12.7	35.8	21.0	66.3	-0.080	-0.245
*I*. *deschampsi*	15,460	34.1	10.7	35.5	19.7	69.6	-0.019	-0.294
*O*. *cordimanus*	15,604	31.8	11.9	34.5	21.8	66.3	-0.043	-0.293
*P*. *tripectinis*	15,612	36.2	10.1	38.0	15.7	74.2	-0.023	-0.215
*M*. *japonicus*	16,170	33.6	10.9	32.8	22.7	66.4	0.014	-0.349
*S*. *neglectum*	15,920	37.4	9.5	38.2	14.9	75.6	-0.010	-0.219
*M*. *depressus*	15765	37.9	8.7	39.4	14.0	77.3	-0.0038	-0.231
*O*. *ceratophthalmus*	15,564	33.7	11.1	35.8	19.4	69.5	-0.029	-0.269
*D*. *pilumnoides*	16,475	37.5	9.5	34.7	18.3	72.2	0.037	-0.316
*M*. *majora*	15,903	38.4	9.8	35.5	16.3	73.9	0.039	-0.248
*H*. *lichuanense*	15,380	35.8	9.3	37.4	17.5	73.2	-0.023	-0.305
*M*. *fornasinii*	15,658	35.5	9.9	36.1	18.5	71.6	-0.0087	-0.303
*C*. *granulosus*	16,300	33.2	11.2	36.1	19.5	69.3	-0.043	-0.272
*M*. *longicarpus*	15,548	32.4	11.8	36.6	19.2	69.0	-0.060	-0.236

### Protein-coding genes

**Fig 2:**
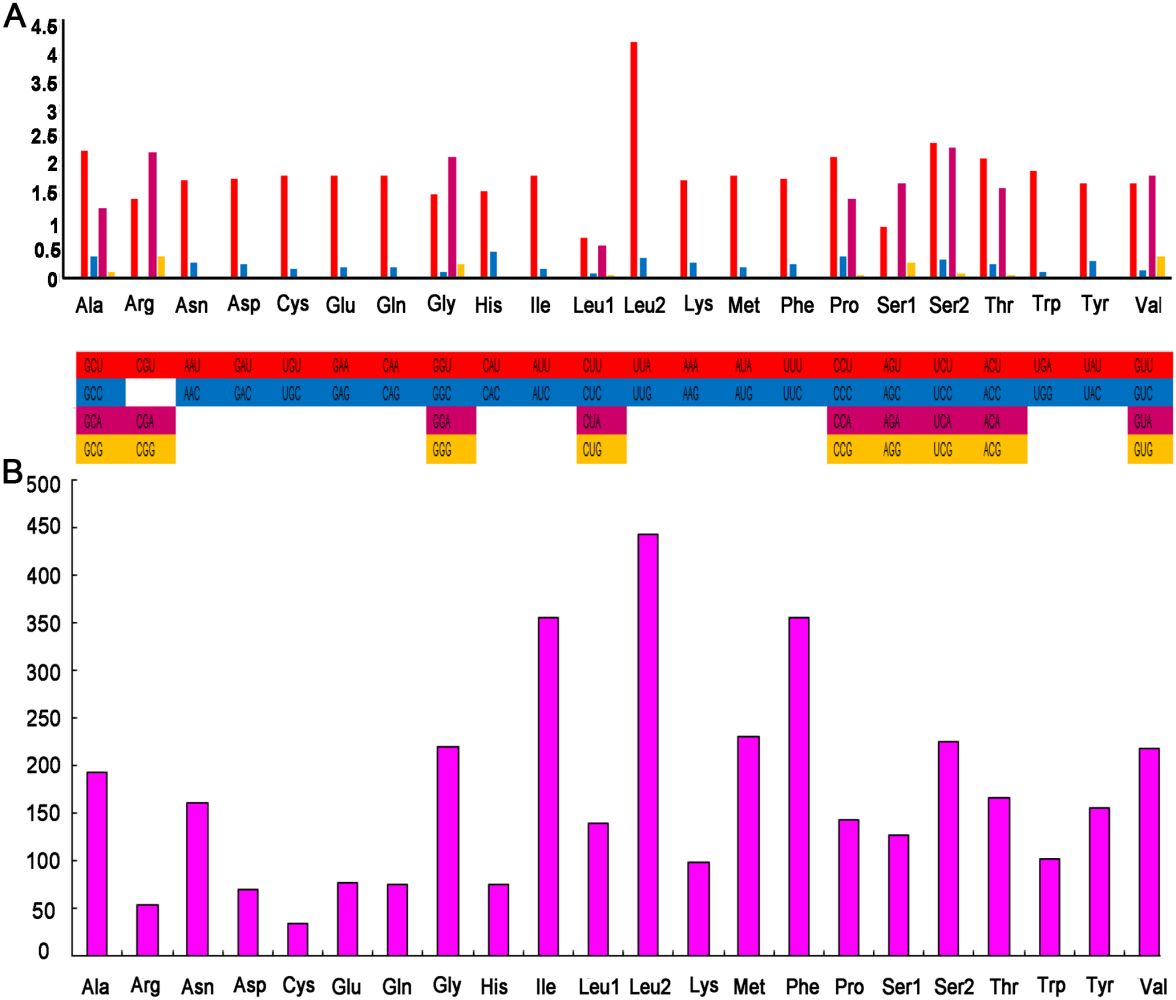
Relative synonymous codon usage (RSCU) in *S*. *sinensi*s mitogenome (A). Amino acid composition in the mitogenome of *S*. *sinensi*s (B).

The mitogenome of *S*. *sinensis* contains 13 PCGs, starting with the typical ATN codons (Table 2). One (*nad1*) starts with ATA, two (*nad3*, *nad6*) with ATT, and ten (*cox1*, *cox2*, *atp8*, *atp6*, *cox3*, *cob*, *nad2*, *nad5*, *nad4*, and *nad4l*) with ATG. Nine PCGs (*cox1*, *cox2*, *atp8*, *nad3*, *nad5*, *nad4*, *nad4l*, *cob*, and *nad1*) have a complete TAA stop codon, while the remaining four terminate with either TA (*atp6*, *cox3*, and *nad6*) or TAG (*nad2*). Nine PCGs (*cox1*, *cox2*, *atp8*, *atp6*, *cox3*, *nad3*, *nad6*, *cob*, and *nad2*) are encoded on the majority strand, while the rest are encoded on the minority strand. The A+T content was 74.0% and AT skew was –0.163 (S1 Table). The RSCU (relative synonymous codon usage) values for *S*. *sinensis* for the third positions is shown in Fig 2A and Table 4. The codon usage is biased: there is a high frequency of AT compared with GC in the third codon position, which is consistent with other crabs. The most common amino acids in mitochondrial proteins are *Leu* (UUR), *Ile* and *Phe* (Fig 2B).

**Table 4:** RSCU (relative synonymous codon usage) of *S*. *sinensis*.

Codon	Count	RSCU	Codon	Count	RSCU	Codon	Count	RSCU	Codon	Count	RSCU
UUU(F)	314	1.76	UCU(S)	106	2.41	UAU(Y)	132	1.69	UGU(C)	32	1.83
UUC(F)	42	0.24	UCC(S)	14	0.32	UAC(Y)	24	0.31	UGC(C)	3	0.17
UUA(L)	408	4.2	UCA(S)	102	2.32	UAA(*)	11	1.83	UGA(W)	98	1.9
UUG(L)	35	0.36	UCG(S)	3	0.07	UAG(*)	1	0.17	UGG(W)	5	0.1
CUU(L)	70	0.72	CCU(P)	77	2.15	CAU(H)	59	1.55	CGU(R)	19	1.41
CUC(L)	9	0.09	CCC(P)	14	0.39	CAC(H)	17	0.45	CGC(R)	0	0
CUA(L)	55	0.57	CCA(P)	50	1.4	CAA(Q)	68	1.81	CGA(R)	30	2.22
CUG(L)	6	0.06	CCG(P)	2	0.06	CAG(Q)	7	0.19	CGG(R)	5	0.37
AUU(I)	325	1.83	ACU(T)	88	2.11	AAU(N)	140	1.74	AGU(S)	40	0.91
AUC(I)	31	0.17	ACC(T)	10	0.24	AAC(N)	21	0.26	AGC(S)	1	0.02
AUA(M)	209	1.81	ACA(T)	67	1.6	AAA(K)	86	1.74	AGA(S)	74	1.68
AUG(M)	22	0.19	ACG(T)	2	0.05	AAG(K)	13	0.26	AGG(S)	12	0.27
GUU(V)	92	1.69	GCU(A)	109	2.26	GAU(D)	63	1.77	GGU(G)	82	1.48
GUC(V)	7	0.13	GCC(A)	19	0.39	GAC(D)	8	0.23	GGC(G)	6	0.11
GUA(V)	99	1.82	GCA(A)	60	1.24	GAA(E)	70	1.82	GGA(G)	119	2.15
GUG(V)	20	0.37	GCG(A)	5	0.1	GAG(E)	7	0.18	GGG(G)	14	0.25

### Transfer RNAs, ribosomal RNAs, and control region

**Fig 3:**
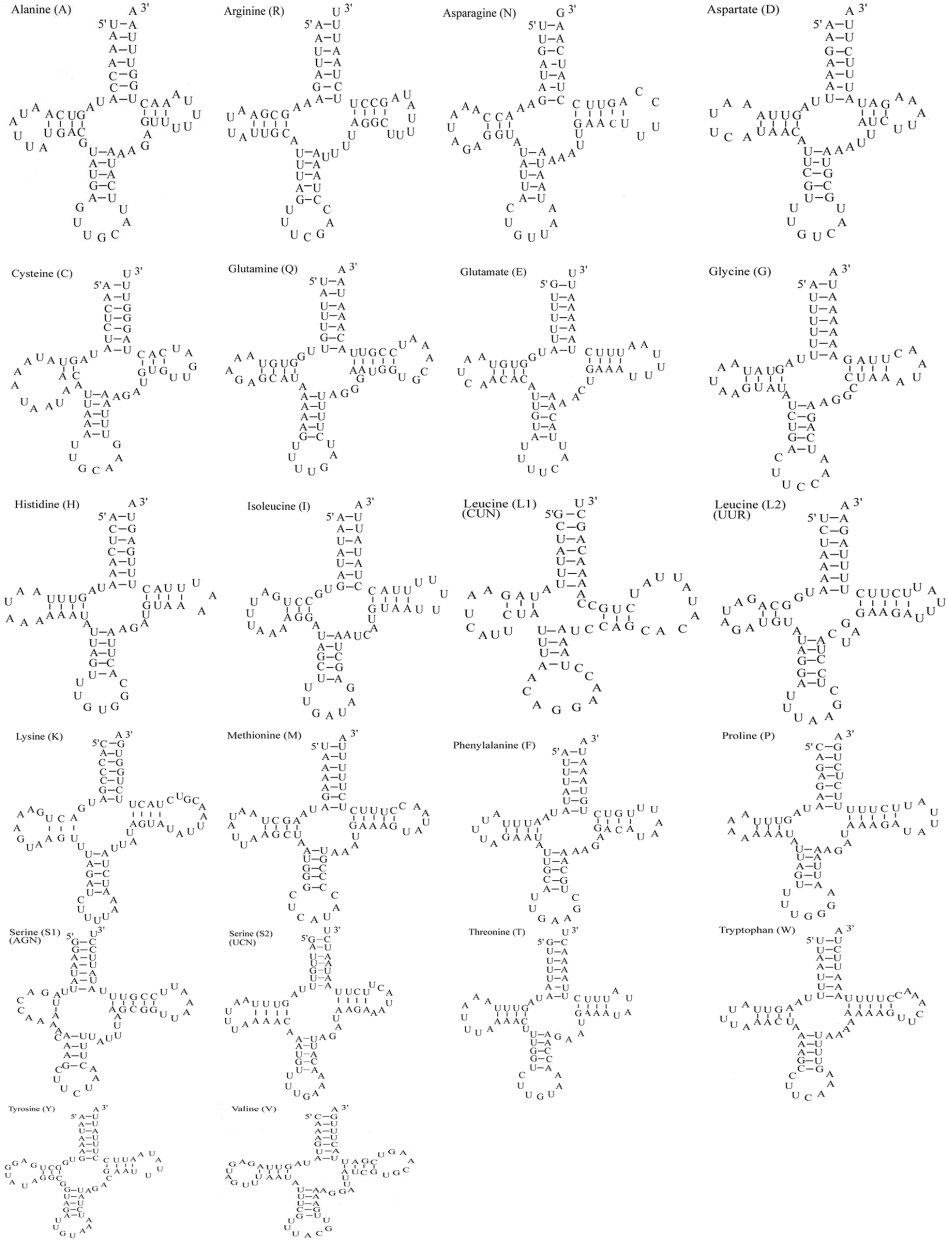
Secondary structures of 22 transfer tRNA genes of *S*. *sinensis*.

The *S*. *sinensis* mitogenome contains 22 tRNA genes, as do most Brachyura mtDNAs. The tRNA genes range from 64 to 73 bp. Fourteen tRNA genes are encoded on the majority strand and eight are encoded on then minority strand (Table 2). All the tRNA genes have a typical cloverleaf structure, except for the *trnS1* gene, whose dihydroxyuridine (DHU) arm had been simplified down to a loop (Fig 3). The loss of the DHU arm is common in animal mitogenomes and has been considered a typical feature of metazoan mitogenomes [30–34]. The cloverleaf secondary structures of 19 transfer RNAs were identified using the web-server of tRNA-scan SE. The three tRNAs not detected by tRNAscan-SE were determined in the unannotated regions by sequence similarity to tRNAs of other crabs. The average AT content of the tRNA genes is 74.6%; their AT skew and GCskew are all negative (S1 Table), showing an obvious bias toward the use of Ts and Cs. Two rRNA genes were identified on the minority strand in the *S*. *sinensis*, with the lrRNA gene located between *trnL* (CUN) and *trnV*, and the srRNA gene located between *trnV* and CR, respectively. The lrRNA gene is 999 bp and the srRNA gene is 822 bp long. The AT-skew of rRNAs (0.001), the GC-skew of rRNAs (–0.296) shows clearly that more As and more Cs than Ts and Gs in rRNAs (S1 Table). CR is located between *rrns* and *trnQ*. The average AT content of the CR is 83.2%. The overall AT-skew and GC-skew in the CR of *S*. *sinensis* are 0.107 and −0.111, respectively (S1 Table), indicating an obvious bias toward the use of As and Cs.

### Gene rearrangement

**Fig 4:**
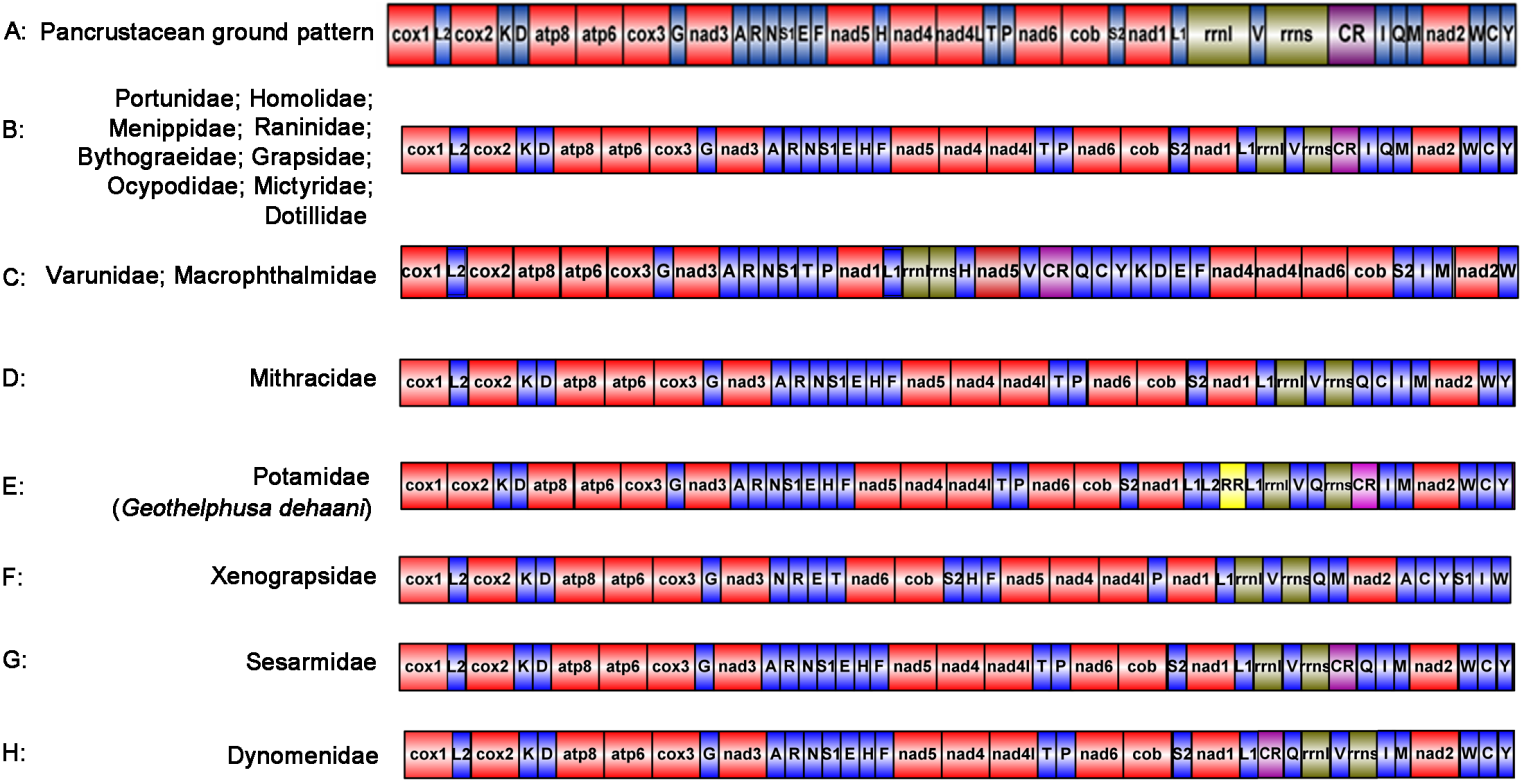
Linearized represantation of gene rearrangements of Brachyura circle mitogeomes. All genes are transcribed from left to right. tRNA genes are exhibited by the corresponding single-letter amino acid code with S1 = AGN, S2 = UCN, L1 = CUN, and L2 = UUR. CR represents control region. *rrnL*, *rrnS*, large and small subunit ribosomal RNA.

In Pancrustaceans, the tRNA gene order between CR and *trnM* is *trnI-trnQ* [35,36] (Fig 4A). The arrangement of the tRNA genes between CR and *trnM* is *trnQ-trnI* in *S*. *sinensis* (Fig 4G). The tRNA rearrangements are generally considered to be a consequence of tandem duplication of part of the mitogenome [37,38]. The arrangement of the tRNA gene between *trnE* and *trnF* is *trnH* in *S*. *sinensis*, which is different from the tRNA genes arrangement of the Pancrustacean ground pattern. In most arthropods, *trnH* is between *nad4* and *nad5* [39], whereas it was found between *trnE* and *trnF* in *S*. *sinensis*. The phenomenon of gene rearrangements in the mitochondrial genome is a relatively common event in crustacean species [40]. The gene order of *S*. *sinensis* is identical to that of *S*. *neglectum* [41], *M*. *depressus* and *P*. *tripectinis* (Fig 4G), which supports the view that *S*. *sinensis* belongs to the Grapsoidea, Sesarmidae. The above results suggested that *S*. *sinensis*, *S*. *neglectum*, *M*. *depressus* and *P*. *tripectinis* are sister groups.

As shown in Fig 4B, the gene orders of these species are identical. The order of the genes in the mitogenome of *S*. *sinensis* is different from that in these Brachyura mitogenomes sequences because of the rearrangement of two tRNA genes between CR and *trnM*. The arrangement of the tRNA genes is *trnQ- trnI* between CR and *trnM* in *S*. *sinensis*, which is different from the *trnI-trnQ* of the these Brachyura species. In this case, the tandem duplication of the gene regions that include *trnI*, *trnQ*, and *trnM*, followed by losses of the supernumerary genes might represent the most ideal mechanism for mitochondrial gene rearrangement [42–44]. It is believed that slipped-strand mispairing takes place first, followed by gene deletion [45]. The gene orders of *Eriocheir japonica sinensis* [46], *E*. *j*. *hepuensis*, *E*. *j*. *japonica*, *Helice latimera*, *Cyclograpsus granulosus* and *M*. *japonicu*s are same (Fig 4C). *H*. *latimera*, *C*. *granulosus*, *E*. *j*. *sinensis*, *E*. *j*. *hepuensis*, and *E*. *j*. *japonica* belong to the Grapoidea, Varunidae [47].

### Phylogenetic analysis

**Fig 5:**
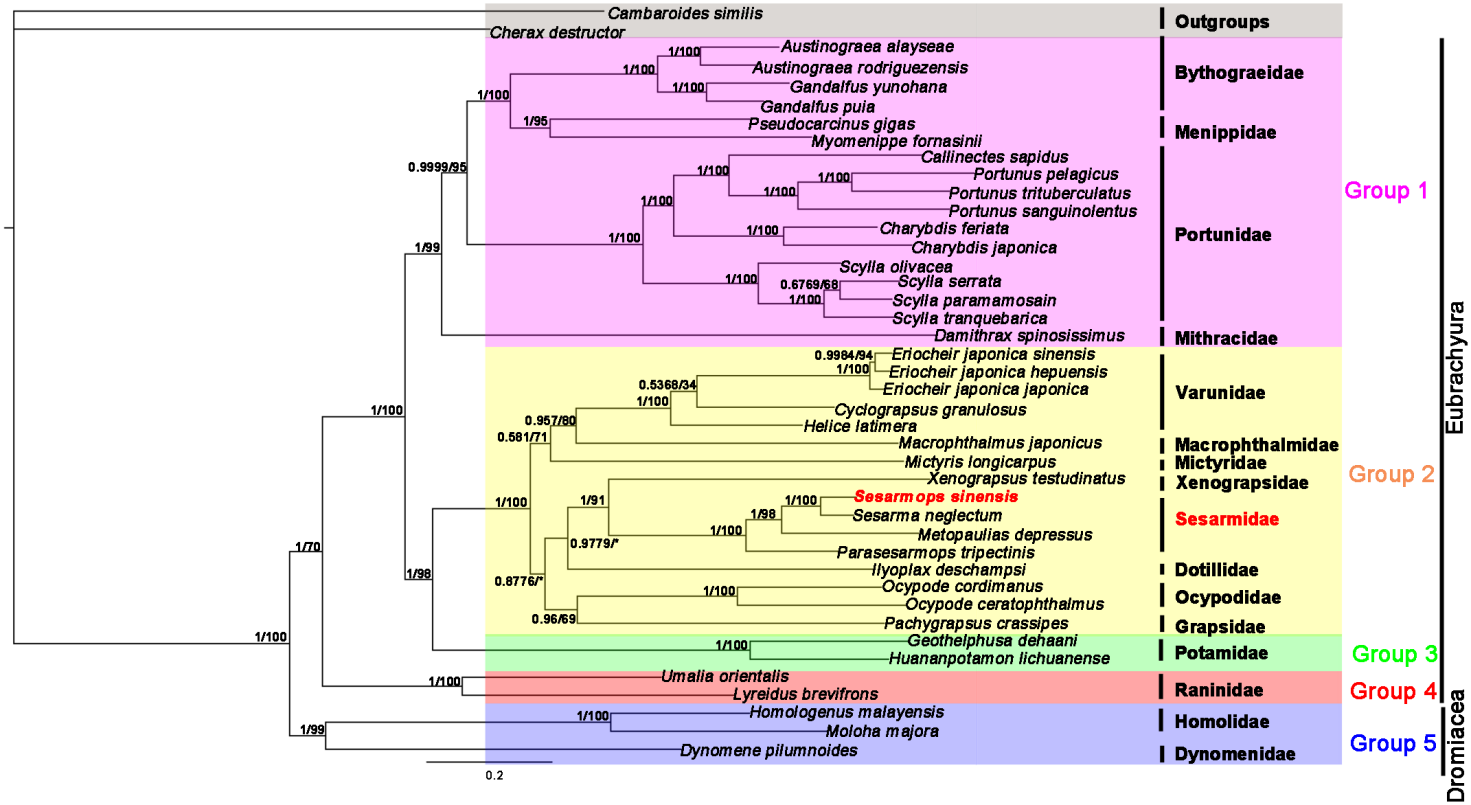
Inferred phylogenetic relationship among Brachyura based on nucleotide sequences of mitochondrial 13PCGs using BI and ML. *C*. *destructor* and *C*. *similis* were used as outgroups.

**Fig 6:**
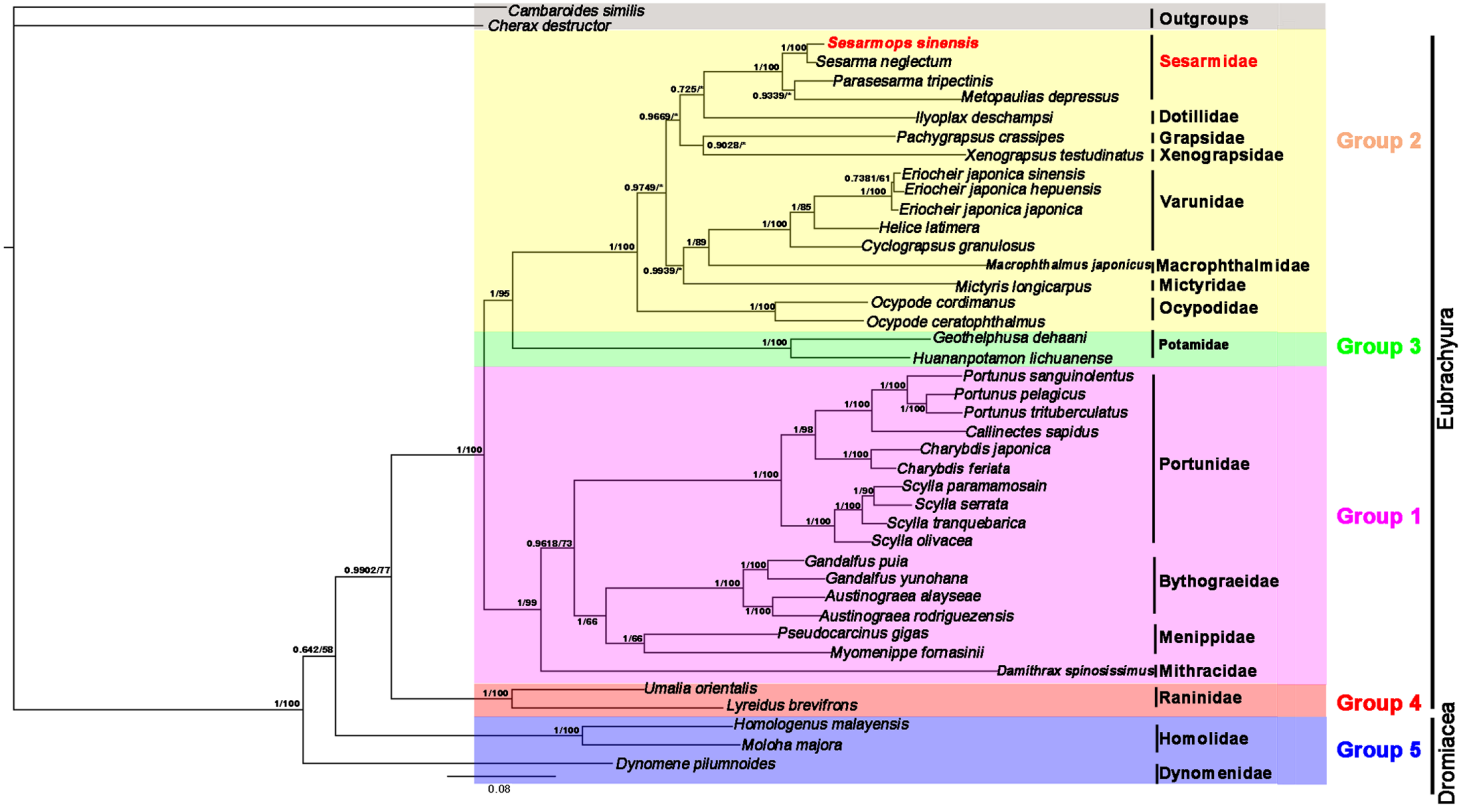
Inferred phylogenetic relationship among Brachyura based on amino acid sequences of mitochondrial 13PCGs using BI and ML. *C*. *destructor* and *C*. *similis* were used as outgroups.

Phylogenetic analyses were based on two datasets: the AA datasets and the NT datasets using two methods (BI and ML) and alignment method of MAFFT, the topologies of phylogenetic analysis in BI and ML were roughly the same, with some slight differences. *S*. *sinensis* and *S*. *neglectum* were clustered in one branch in the phylogenetic tree with high nodal support values in BI and ML trees. (*S*. *sinensis + S*. *neglectum*) clade is well supported to be the sister group to the (*P*. *tripectinis* + *M*. *depressus*) clade. This supported the view that *S*. *sinensis* belongs to the Grapoidea, Sesarmidae, which is consistent with the results of the gene rearrangement analysis. *H*. *latimera*, *C*. *granulosus*, *E*. *j*. *sinensis*, *E*. *j*. *hepuensis*, and *E*. *j*. *japonica* clustered together with high statistical support (Figs 5 and 6), showing that these species have sister group relationships and belong to Grapsoidea, Varunidae. *P*. *crassipes* belong to the Grapoidea, Grapsidae [48]. Previous studies noted an ambiguous classification for *Ilyoplax deschampsi*. *I*. *deschampsi* is part of the Dotillidae in this ambiguous classification. The phylogenetic position of *I*. *deschampsi* is within the Grapsoidea [2,49,50]. The real phylogenetic position of *I*. *deschampsi* should be closer to the Grapsoidea species that shown in Figs 5 and 6. *Xenograpsus testudinatus*, originally placed in the Varunidae, has been transferred to its own family (Xenograpsidae) [51].

BI and ML trees of the nucleotide sequences and amino acid sequences of mitochondrial 13 PCGs (Figs 5 and 6) with *C*. *destructor* and *C*. *similis* as outgroups generated identical tree topologies. The section Eybrachyura crabs consist of four groups, one comprising famlies of Heterotremata, the second Thoracotremata famlies, the third Potamoidea crabs, and the fourth Raninoida species. All the bootstrap values for the branches separating these groups are high. The resulting phylogeny supports the establishment of a separate subsection Potamoida corresponding to Group 3. The present molecular study gives additional evidence for the Potamoida status in these taxa. In all trees Potamoida does closely cluster with subsection Thoracotremata. The relationship between Potamoida and Thoracotremata is much closer than it between the former and Heterotremata, which was proposed by Bowman and Abele [4]. These four subsections (groups) constitute a monophyletic sister group to Section Dromiacea (Group 5) in all phylogenetic trees.

## Conclusion

This study presents one mitogenome of *S*. *sinensis*. The mitogenome contains 13 PCGs, 22 tRNA genes, two rRNA genes and CR. The AT skew and the GC skew are both negative in the mitogenomes of *S*. *sinensis*, indicating an obvious bias toward the use of Ts and Cs, which is consistent with most sequenced brachyuran crabs. The gene arrangement of *S*. *sinensis* is identical to that of *S*. *neglectum*, *P*. *tripectinis* and *M*. *depressus*. In comparison to Pancrustacean ground pattern and common arrangement for brachyuran crabs, *S*. *sinensis* exhibits a novel rearrangement. Tandem duplication followed by random deletion is widely considered to explain generation of gene rearrangement of mitogenome in *S*. *sinensis*. The phylogenetic analyses indicate that *S*. *sinensis* and *S*. *neglectum* have sister group relationships, and the clade (*S*. *sinensis + S*. *neglectum*) is well supported to be the sister group to (*P*. *tripectinis* + *M*. *depressus*), suggesting that *S*. *sinensis* should belong to Grapoidea, Sesarmidae. The topology of BI and ML trees of Brachyura species (Fig 5) inferred from nucleotide sequences of mitochondrial 13 PCGs sequences are similar to those of Fig 6 constructed from amino acid sequences of whole mitogenomes. The resulting phylogeny strongly supports the establishment of a separate subsection Potamoida, so section Eubrachyura consists of four subsections which are Raninoida, Heterotremata, Potamoida, and Thoracotremata. However, the four subsections and two sections are monophyletic, respectively, whereas the relationships within families of each subsection were not resolved absolutely in the present study.

## Supporting information

S1 Table Composition and skewness in the *S*. *sinensis* mitogenome. (DOCX)

## References

[1] Guinot DM, Tavares M, Castro P. Significance of the sexual openings and supplementary structures on the phylogeny of brachyuran crabs (Crustacea, Decapoda, Brachyura), with new nomina for higher ranked podotreme taxa. Zootaxa. 2013; 3665: 1–414. 26401537 10.11646/zootaxa.3665.1.1

[2] Ji YK, Wang A, Lu XL, Song DH, Jin YH, Lu JJ, et al Mitochondrial genomes of two brachyuran crabs (Crustacea: Decapoda) and phylogenetic analysis. J Crustacean Biol. 2014; 34: 494–503.

[3] Guinot D. Propositions pour une nouvelle classification des Crustacés Décapodes Brachyoures. Comptes Rendus Hebdomadaires des Séances de l’Académie des Sciences. 1977; 285: 1049–1052.

[4] Bowman TE, Abele LG. Classification of the Recent Crustacea. In Systematics, the fossil record, and biogeography, ed. Abele L. G., vol. I of The biology of Crustacea, ed. Bliss D. E.. New York: Academic Press 1982; 1–27.

[5] Martin JW, Davis GE. An updated Classification of the Recent Crustacea. Natural History Museum of Los Angeles County. 2001; 39: 1–124.

[6] Ng PKL, Guinot D, Davie PJF. Systema Brachyuorum: part I. An annotated checklist of extant Brachyuran crabs of the world. Raffles B Zool. 2008; 17: 1–286.

[7] Yang SL, Chen HL, Dai AY. Fauna sinica invertebrata Vol.49 Crustacea Decapoda Portunidae. Beijing: Science Press 2012; 396–417.

[8] Boore JL. Animal mitochondrial genomes. Nucleic Acids Res. 1999; 27: 1767–1780. 10101183 10.1093/nar/27.8.1767 PMC148383

[9] Zhang S, Li XL, Cui ZX, Wang HX, Wang CL, Liu XL. The application of mitochondrial DNA in phylogeny reconstruction and species identification of portunid crab. Mar Sci. 2008; 32: 9–18.

[10] Dabney J, Knapp M, Glocke I, Gansauge MT, Weihmann A, Nickel B, et al Complete mitochondrial genome sequence of a Middle Pleistocene cave bear reconstructed from ultrashort DNA fragments. Proc Natl Acad Sci USA. 2013; 110: 15758–15763. doi: 10.1073/pnas.1314445110 24019490 10.1073/pnas.1314445110 PMC3785785

[11] Zhang P, Liang D, Mao RL, Hillis DM, Wake DB, Cannatella DC. Efficient sequencing of Anuran mtDNAs and a mitogenomic exploration of the phylogeny and evolution of frogs. Mol Biol Evol. 2013; 30: 1899–1915. doi: 10.1093/molbev/mst091 23666244 10.1093/molbev/mst091

[12] Sun HY, Zhou KY, Song DX. Mitochondrial genome of the Chinese mitten crab *Eriocheir japonica sinenesis* (Brachyura: Thoracotremata: Grapsoidea) reveals a novel gene order and two target regions of gene rearrangements. Gene. 2005; 349: 207–217. doi: 10.1016/j.gene.2004.12.036 15780981 10.1016/j.gene.2004.12.036

[13] Roe AD, Sperling FAH. Patterns of evolution of mitochondrial cytochrome c oxidase I and II DNA and implications for DNA barcoding. Mol Phylogenet Evol. 2007; 44: 325–345. doi: 10.1016/j.ympev.2006.12.005 17270468 10.1016/j.ympev.2006.12.005

[14] Wolstenholme DR. Animal mitochondrial DNA: structure and evolution. Int Rev Cytol. 1992; 141: 173–216. 1452431 10.1016/s0074-7696(08)62066-5

[15] Yang JS, Nagasawa H, Fujiwara Y. The complete mitogenome of the hydrothermal vent crab *Gandalfus yunohana* (Crustacea: Decapoda: Brachyura): a link between the Bythograeoidea and Xanthoidea. Zool Scr. 2010; 39: 621–630.

[16] Shi G, Cui Z, Hui M. The complete mitochondrial genomes of *Umalia orientalis* and *Lyreidus brevifrons*: The phylogenetic position of the family Raninidae within Brachyuran crabs. Mar Genom. 2015; 21: 53–61. 10.1016/j.margen.2015.02.002 25744934

[17] Tan MH, Gan HM, Lee YP. The complete mitogenome of the swimming crab *Thalamita crenata* (Rüppell, 1830) (Crustacea; Decapoda; Portunidae). Mitochondr DNA. 2014; 27: 1275–1276. 10.3109/19401736.2014.945553 25090400

[18] Abele LG. Comparison of morphological and molecular phylogeny of the Decapoda. Mem Queensl Mus. 1991; 31: 101–108.

[19] Katoh K, Misawa K, Kuma K. MAFFT: a novel method for rapid multiple sequence alignment based on fast Fourier transform. Nucleic Acids Res. 2002; 30: 3059–3066. 12136088 10.1093/nar/gkf436 PMC135756

[20] Castresana J. Selection of conserved blocks from multiple alignments for their use in phylogenetic analysis. Mol Biol Evol. 2000; 17: 540–552. 10742046 10.1093/oxfordjournals.molbev.a026334

[21] Xia X, Xie Z. DAMBE: software package for data analysis in molecular biology and evolution. J Hered. 2001; 92: 371–373. 11535656 10.1093/jhered/92.4.371

[22] Lowe TM, Eddy SR. tRNAscan-SE: a program for improved detection of transfer RNA genes in genomic sequence. Nucleic Acids Res. 1997; 25: 955–964. 9023104 10.1093/nar/25.5.955 PMC146525

[23] Ronquist F, Huelsenbeck JP, Teslenko M. Draft MrBayes version 3.2 Manual. Tutorials and Model Summaries. 2011.

[24] Silvestro D, Michalak I. raxmlGUI: a graphical front-end for RAxML. Org Divers Evol. 2012; 12: 335–337.

[25] Cui BK, Tang LP, Dai YC. Morphological and molecular evidences for a new species of Lignosus (Polyporales, Basidiomycota) from tropical China. Myco Prog. 2011; 10: 267–271.

[26] Abascal F, Zardoya R, Posada D. ProtTest: selection of best-fit models of protein evolution. Bioinformatics. 2005; 21: 2104–2105. doi: 10.1093/bioinformatics/bti263 15647292 10.1093/bioinformatics/bti263

[27] Stamatakis A, Hoover P, Rougemont J. A rapid bootstrap algorithm for the RAxML web-servers. Syst Biol. 2008; 57: 758–771. doi: 10.1080/10635150802429642 18853362 10.1080/10635150802429642

[28] Rokas A, Williams BL, King N, Carroll SB. Genome-scale approaches to resolving incongruence in molecular phylogenies. Nature. 2003; 425: 798–804. doi: 10.1038/nature02053 14574403 10.1038/nature02053

[29] Perna NT, Kocher TD. Patterns of nucleotide composition at fourfold degenerate sites of animal mitochondrial genomes. J Mol Evol. 1995; 41: 353–358. 7563121 10.1007/BF00186547

[30] Liu Y, Cui ZX. Complete mitochondrial genome of the Asian paddle crab *Charybdis japonica* (Crustacea: Decapoda: Portunidae): gene rearrangement of the marine brachyurans and phylogenetic considerations of the decapods. Mol Biol Rep. 2010; 37: 2559–2569. doi: 10.1007/s11033-009-9773-2 19714481 10.1007/s11033-009-9773-2

[31] Ma HY, Ma CY, Li XC, Xu Z, Feng NN, Ma LB. The complete mitochondrial genome sequence and gene organization of the mud crab (*Scylla paramamosain*) with phylogenetic consideration. Gene. 2013; 519: 120–127. doi: 10.1016/j.gene.2013.01.028 23384716 10.1016/j.gene.2013.01.028

[32] Ma HY, Ma CY, Li CH, Lu JX, Zou X, Gong YY, et al First mitochondrial genome for the red crab (*Charybdis feriata*) with implication of phylogenomics and population genetics. Sci Rep. 2015; 5:10.1038/srep11524 PMC4520191 26225473

[33] Ki JS, Dahms HU, Hwang JS. The complete mitogenome of the hydrothermal vent crab *Xenograpsus testudinatus* (Decapoda, Brachyura) and comparison with brachyuran crabs. Comp Biochem Phys D. 2009; 4: 290–299. 10.1016/j.cbd.2009.07.002 20403751

[34] Liu QN, Zhang HB, Jiang SH, Xuan FJ, Li CF, Zhang DZ, et al The complete mitochondrial genome of *Eriocheir japonica sinensis* (Decapoda: Varunidae) and its phylogenetic analysis. Biochem Sys Ecol. 2015; 62: 241–248.

[35] Boore JL, Lvaroa DV, Brown WM. Gene translocation links insects and crustaceans. Nature. 1998; 392: 667–668. doi: 10.1038/33577 9565028 10.1038/33577

[36] Regier JC, Shultz JW, Kambic RE. Pancrustacean phylogeny: hexapods are terrestrial crustaceans and maxillopods are not monophyletic. P Roy Soc Lond B Bio. 2005; 272: 395–401. 10.1098/rspb.2004.2917 PMC1634985 15734694

[37] Juhling F, Putz J, Bernt M, Donath A, Middendorf M, Florentz C, et al Improved systematic tRNA gene annotation allows new insights into the evolution of mitochondrial tRNA structures and into the mechanisms of mitochondrial genome rearrangements. Nucleic Acids Res. 2012; 40: 2833–2845. doi: 10.1093/nar/gkr1131 22139921 10.1093/nar/gkr1131 PMC3326299

[38] Gong YJ, Shi BC, Kang ZJ, Zhang F, Wei SJ. The complete mitochondrial genome of the oriental fruit moth *Grapholita molesta* (Busck) (Lepidoptera: Tortricidae). Mol Biol Rep. 2012; 39: 2893–2900. doi: 10.1007/s11033-011-1049-y 21670960 10.1007/s11033-011-1049-y PMC3271229

[39] Podsiadlowski L, Braband A. The complete mitochondrial genome of the sea spider *Nymphon gracile* (Arthropoda: Pycnogonida). BMC genomics. 2006; 7: 1. 17087824 10.1186/1471-2164-7-284 PMC1636051

[40] Shen X, Ren JF, Cui ZX, Sha ZL, Wang B, Xiang JH, et al The complete mitochondrial genomes of two common shrimps (*Litopenaeus vanname*i and *Fenneropenaeus chinensis*) and their phylogenomic considerations. Gene. 2007; 403: 98–109. doi: 10.1016/j.gene.2007.06.021 17890021 10.1016/j.gene.2007.06.021

[41] Xing YH, Ma XP, Wei YQ, Pan D, Liu WL, Sun HY. The complete mitochondrial genome of the semiterrestrial crab, *Chiromantes neglectum* (Eubrachyura: Grapsoidea: Sesarmidae). Mitochondr DNA. 2016; 1: 461–463. 10.1080/23802359.2016.1186509 PMC7799694 33473520

[42] Cao YQ, Ma C, Chen JY, Yang DR. The complete mitochondrial genomes of two ghost moths, *Thitarodes renzhiensis* and *Thitarodes yunnanensis*: the ancestral gene arrangement in Lepidoptera. BMC genomics. 2012; 13: 1. 22726496 10.1186/1471-2164-13-276 PMC3463433

[43] Rawlings TA, Collins TM, Bieler R. Changing identities: tRNA duplication and remolding within animal mitochondrial genomes. P Natl Acad Sci. 2003; 100: 15700–15705. 10.1073/pnas.2535036100 PMC307631 14673095

[44] Shi W, Gong L, Wang SY. Tandem duplication and random loss for mitogenome rearrangement in Symphurus (Teleost: Pleuronectiformes). BMC genomics. 2015; 16: 1. 25943439 10.1186/s12864-015-1581-6 PMC4430869

[45] Yamauchi MM, Miya M, Nishida M. Complete mitochondrial DNA sequence of the swimming crab, *Portunus trituberculatus* (Crustacea: Decapoda: Brachyura). Gene. 2003; 311: 129–135. 12853147 10.1016/s0378-1119(03)00582-1

[46] Tang BP, Zhou KY, Song DX, Yang G, Dai AY. Molecular systematics of the Asian mitten crabs, genus *Eriocheir* (Crustacea: Brachyura). Mol phylogenet Evol. 2003; 29: 309–316. 13678686 10.1016/s1055-7903(03)00112-x

[47] Komai T, Yamasaki I, Kobayashi S, Yamamoto T, Watanabe S. Eriocheir ogasawaraensis Komai, a new species of mitten crab (Crustacea: Decapoda: Brachyura: Varunidae) from the Ogasawara Islands, Japan, with notes on the systematics of Eriocheir De Haan, 1835. Zootaxa. 2006; 1168: 1–20.

[48] Cassone BJ, Boulding EG. Genetic structure and phylogeography of the lined shore crab, *Pachygrapsus crassipes*, along the northeastern and western Pacific coasts. Mar Biol. 2006; 149: 213–226.

[49] Schubart CD, Neigel JE, Felder DL. Use of the mitochondrial 16S rRNA gene for phylogenetic and population studies of Crustacea. Crustacean issues. 2000; 12: 817–830.

[50] Kitaura J, Wada K, Nishida M. Molecular phylogeny of grapsoid and ocypodoid crabs with special reference to the genera *Metaplax* and *Macrophthalmus*. J Crustacean Biol. 2002; 22: 682–693.

[51] Ng NK, Davie PJF, Schubart CD. Xenograpsidae, a new family of crabs (Crustacea: Brachyura) associated with shallow water hydrothermal vents. Raffles B Zool. 2007; 16 Suppl: 233–256.

