# Supplementary material for: The complete mitochondrial genome of Sesarmops sinensis reveals gene rearrangements and phylogenetic relationships in Brachyura
Source: PLoS One. 2017 Jun 16;12(6):e0179800. doi: 10.1371/journal.pone.0179800 (PMC5473591; doi:10.1371/journal.pone.0179800)
Supplement: S1 Table — (DOCX) [file pone.0179800.s001.docx]

S1 Table Composition and skewness in the *S. sinensis* mitogenome.

| *S. sinensis* | Size (bp) | A(bp) | tCT(bp) | G(bp) | C(bp) | A % | T% | G% | C% | AT% | AT skew | GC skew |
| --- | --- | --- | --- | --- | --- | --- | --- | --- | --- | --- | --- | --- |
| Whole genome | 15,905 | 5944 | 616093 | 1493 | 2375 | 37.4 | 38.3 | 9.4 | 14.9 | 75.7 | -0.012 | –0.228 |
| 13 Protein-coding genes | 11,179 | 3461 | 4809 | 1472 | 1437 | 31.0 | 43.0 | 13.2 | 12.8 | 74.0 | –0.163 | 0.012 |
| tRNA genes | 1478 | 548 | 555 | 161 | 214 | 37.1 | 37.5 | 10.9 | 14.5 | 74.6 | –0.006 | –0.141 |
| rRNA genes | 1821 | 724 | 722 | 132 | 243 | 39.8 | 39.6 | 7.3 | 13.3 | 79.4 | 0.001 | –0.296 |
| Control region | 751 | 346 | 279 | 56 | 70 | 46.1 | 37.1 | 7.5 | 9.3 | 83.2 | 0.107 | –0.111 |
